# Applications of Raman Spectroscopy in Bacterial Infections: Principles, Advantages, and Shortcomings

**DOI:** 10.3389/fmicb.2021.683580

**Published:** 2021-07-19

**Authors:** Liang Wang, Wei Liu, Jia-Wei Tang, Jun-Jiao Wang, Qing-Hua Liu, Peng-Bo Wen, Meng-Meng Wang, Ya-Cheng Pan, Bing Gu, Xiao Zhang

**Affiliations:** ^1^Institute Pasteur of Shanghai, Chinese Academy of Sciences, Shanghai, China; ^2^Department of Bioinformatics, School of Medical Informatics and Engineering, Xuzhou Medical University, Xuzhou, China; ^3^State Key Laboratory of Quality Research in Chinese Medicines, Macau University of Science and Technology, Taipa, China; ^4^Jiangsu Key Laboratory of New Drug Research and Clinical Pharmacy, School of Pharmacy, Xuzhou Medical University, Xuzhou, China; ^5^School of Life Sciences, Xuzhou Medical University, Xuzhou, China; ^6^Laboratory Medicine, Guangdong Provincial People’s Hospital, Guangdong Academy of Medical Sciences, Guangzhou, China

**Keywords:** Raman spectroscopy, bacterial pathogen, machine learning, infectious disease, antibiotic resistance

## Abstract

Infectious diseases caused by bacterial pathogens are important public issues. In addition, due to the overuse of antibiotics, many multidrug-resistant bacterial pathogens have been widely encountered in clinical settings. Thus, the fast identification of bacteria pathogens and profiling of antibiotic resistance could greatly facilitate the precise treatment strategy of infectious diseases. So far, many conventional and molecular methods, both manual or automatized, have been developed for *in vitro* diagnostics, which have been proven to be accurate, reliable, and time efficient. Although Raman spectroscopy (RS) is an established technique in various fields such as geochemistry and material science, it is still considered as an emerging tool in research and diagnosis of infectious diseases. Based on current studies, it is too early to claim that RS may provide practical guidelines for microbiologists and clinicians because there is still a gap between basic research and clinical implementation. However, due to the promising prospects of label-free detection and noninvasive identification of bacterial infections and antibiotic resistance in several single steps, it is necessary to have an overview of the technique in terms of its strong points and shortcomings. Thus, in this review, we went through recent studies of RS in the field of infectious diseases, highlighting the application potentials of the technique and also current challenges that prevent its real-world applications.

## Introduction

Infections caused by bacterial pathogens in clinical settings are commonly encountered, which is considered as the top 10 most common causes of death globally (Abayasekara et al., 2017). Acute bacterial infections could be serious or even deadly, especially when bacteria enter into bloodstream or cross the blood–brain barrier (van Sorge and Doran, 2012). In addition, antibiotic resistance plays important roles in bacterial pathogenicity during host infection. Thus, rapid detection of bacterial infection and profiling of drug resistance are crucial in guiding effective treatments of infectious diseases (Burnham et al., 2017). Conventional methods for bacterial diagnosis, such as medium culture, biochemical reactions, and serological tests are well-established techniques with high reliability and accuracy. However, some of these techniques are laborious, costly, and time consuming, which also have comparatively steep learning curves for real-world use (Franco-Duarte et al., 2019). Thus, new diagnostic methods have been developed for rapid and minimally invasive detection of bacterial pathogens in order to meet clinical requirements or investigate infectious disease outbreaks (Fournier et al., 2014), such as polymerase chain reaction (PCR), enzyme-linked immunosorbent assay (ELISA), high-throughput next-generation sequencing (NGS), and chemical analysis methods like mass spectrometry (MS). In recent years, Raman spectroscopy (RS) is gaining more and more attentions in research fields and in clinical settings due to advancements in instrumentation and data-handling techniques (Wang et al., 2016). As an easy-to-learn, low-cost, and label-free chemical analysis technique, RS has both great potentials and huge challenges in clinical pathogen analysis (Sil et al., 2020), which drives researchers to work hard in the field to bridge the gap between experimental setup and clinical implementation. In this review, we focus on the principles, advantages, and shortcomings of the RS technology in a concise manner, highlighting the application potentials of the technique and also current challenges that prevent its real-world applications.

## Conventional and Molecular Methods

Conventionally, the detection of bacterial pathogens in clinical infection relies on methods like medium culture (e.g., colony size, color, and shape), microscopy (e.g., Gram stain), biochemical analysis (catalase activity, oxidase activity, and urease activity, etc.), and serological tests (e.g., latex agglutination tests; Váradi et al., 2017). The presence of antibiotic resistance adds more complexity during the identification of bacterial infections. Classical methods for antibiotic susceptibility testing (AST) include but not limited to disk diffusion, Epsilometer test (*E*-test), and microdilution, which also require medium culture (Khan et al., 2019). However, only a small number of bacteria could be successfully cultured due to the fastidious growth requirement, which makes accurate diagnosis of bacterial infection a challenge.

The development of molecular diagnostic methods greatly improves bacterial identification and antibiotics profiling, which mainly relies on the analysis of genomic markers corresponding to nucleic acid sequences (Váradi et al., 2017). For example, PCR is a fast and reliable molecular method for the identification of bacterial infections, which directly detects bacterial pathogens by genetic materials and requires primers for the amplification process (Barghouthi, 2011). One of the advantages of PCR is its capacity in recognizing bacterial infection at early stage when no sufficient antibodies against the pathogens are produced (Kubina and Dziedzic, 2020). However, once the pathogens are cleared from the immune system or become dormant, no genetic materials could be detected. Thus, PCR is better to be used during the acute infection stage and cannot be used for retrospective analysis. In recent years, universal primers with the capacity of identifying highly conservative regions of genes like 16s rDNA have also been widely used to find previously unrecognized or uncultured organisms from infected host tissues, leading to the characterization of microbial diversity within a sample, which is also known as metagenomics (Abayasekara et al., 2017).

Enzyme-linked immunosorbent assay is a type of immunosorbent assay that can be used for bacterial identification through detecting the presence of antigens or antibodies in blood sera. In the food industry, ELISA is one of the most commonly used immunological methods for foodborne pathogen detections (Law et al., 2015). Recently, some comparative studies indicated that ELISA had great potential in clinical applications due to its superiority to conventional methods in the diagnosis of bacterial infections (Xu et al., 2020). However, antibody levels in the early stage of post-infection may not be reliably detectable. Currently, ELISA has not been used in routine bacterial diagnostics, which may be due to its limitations such as high costs, poor reproducibility, high false-positive rates, and antibody instability (Sakamoto et al., 2017). One advantage of the serological testing via ELISA method, when compared with other methods based on genetic materials, is that it is able to study the epidemiology of diseases in different populations retrospectively due to the persistence of antibodies in the bloodstream after microbial infections (Lai et al., 2020).

As for the sequencing technology, it used to be difficult to access but is now affordable in microbial studies due to the fast development of instruments (Kwong et al., 2015). For example, NGS and long-read sequencing could provide high-resolution discrimination of bacterial pathogens at nucleic acids level, which could reliably distinguish closely related bacterial lineages and accurately track the outbreaks (Balloux et al., 2018; Logsdon et al., 2020). In addition, through comparative genome analysis, gain or loss of particular genes could be used to predict specific phenotypes such as stress resistance and pathogenicity (Stratakos et al., 2019), while genome-wide association study could reveal antibiotic resistance (Lees et al., 2020). As for microbial composition in a clinical sample from mouth, skin, or gut, metagenomic next-generation sequencing plays a pivotal role, which greatly facilitates the understanding of antimicrobial resistance, microbiome, human host gene expression, and oncology (Chiu and Miller, 2019). Although NGS provides an overview of bacterial species at genomic level with high accuracy, sequencing is still far away to be a routine method because of the high costs, labor intensity, complex sample preparation steps, and sophisticated data analysis procedures (Deurenberg et al., 2017). Currently, the application of NGS methods is mainly limited to laboratory experiments and epidemiological investigations while being rarely used for routine microbial identification or susceptibility testing in clinical laboratories (Deurenberg et al., 2017; Rossen et al., 2018).

## Chemical Analysis Methods

### Mass Spectroscopy

In recent years, chemical analysis via precision instruments is getting more and more attention from both industrial, clinical, and academic fields, among which MS is an important analytical tool due to its high-throughput capacity, sensitivity, and specificity (Sauer and Kliem, 2010). Although several MS methods, together with software tools, have been developed, matrix-assisted laser desorption/ionization time-of-flight mass spectrometry (MALDI-TOF MS) is one of the most popular MS instruments used in clinical microbiology due to the rapid and accurate identification of an extensive range of bacteria (Hou et al., 2019). In specificity, MALDI-TOF MS is an inexpensive and straightforward method for bacterial classification and identification on genus, species, and, sometimes, subspecies level (Sauer and Kliem, 2010). In addition, databases containing MS spectra of known organisms provide much more convenience in the identification of species with similar phenotypic, genotypic, and biochemical properties (Singhal et al., 2015). However, there are also some limitations for MALDI-TOF MS. For example, it is difficult for MALDI-TOF MS to discriminate closely related bacterial species such as *Escherichia coli* and *Shigella*. It is also hard for MALDI-TOF MS to differentiate some antibiotic resistance phenotypes, such as methicillin-resistant and methicillin-sensitive *Staphylococcus aureus* (Florio et al., 2018).

### Raman Spectroscopy

Raman spectroscopy is an emerging method for the identification of bacterial infections because it can act as a rapid, efficient, and minimally invasive tool to identify bacterial cells and antibiotic resistance, which also has the potentials in high-throughput and real-time applications in the field of clinical diagnostics (Strola et al., 2014). The basic principle of the Raman effect is that when the smallest unit of light passes through any medium, the light scattered by other molecules affects the frequency change, which means the Raman effect is caused by the vibration of molecules and thus can be explained by energy levels (Jones et al., 2019). From the perspective of quantum mechanics, the Raman effect is the inelastic collision that occurs when photons collide with molecules. If the molecule is at the ground state level at the beginning, and then when the excited light interacts with the molecule, the molecule will be excited to a high energy level or virtual state, and then, the molecules and electrons in the virtual state will transition to the excited state, generating scattered light. In this process, energy will be transferred to the molecule by the excited photon, while the photon loses its energy in this process. At this time, the molecule that transitions to the excited state gains energy.

**FIGURE 1:**
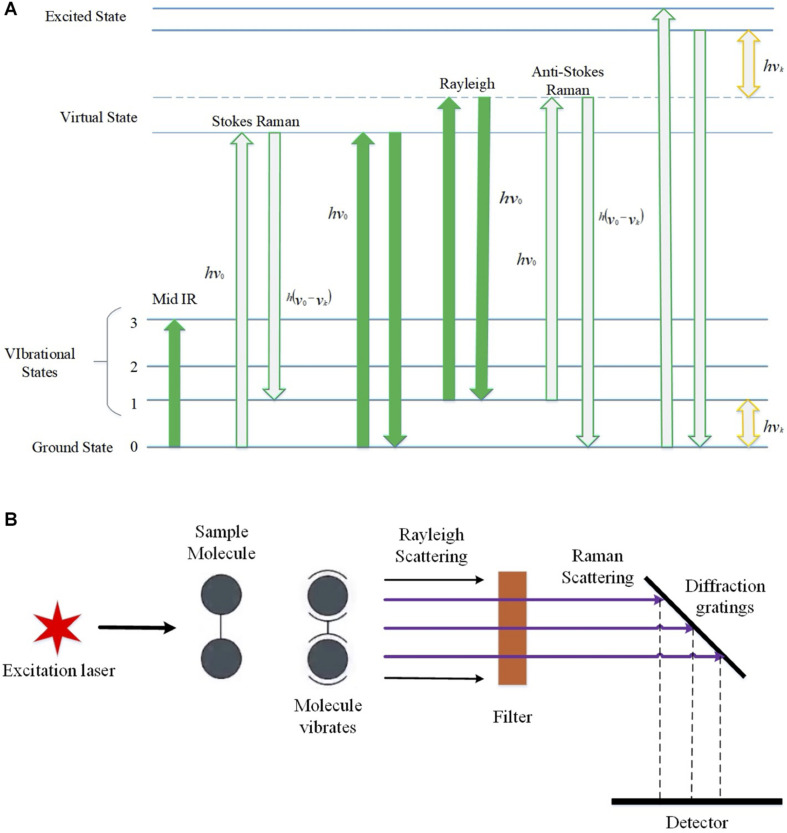
Schematic illustration of the basic principles of Raman effects and the brief architecture of Raman spectroscopy. **(A)** Raman spectrum energy level diagram, which shows the transition process of infrared light irradiation, Stokes rays, anti-Stokes rays, Rayleigh scattering, and Raman scattering. *hv_k_*, initial irradiation energy; *E*_0_, ground state; *E*_1_, vibration excited state; *E*_0_+*hv*_0_ and *E*_1_+*hv*_0_, excited virtual state. **(B)** Schematic diagram of Raman spectroscopy. After the incident light is irradiated, the molecules reach an excited state. The light of different frequencies during the scattering process is Raman scattering, which is reflected on the grating and captured by the detector.

There are some positions where the incident light frequency is at low level, and at these positions, the accepted scattered light is called Stokes Line, while, on the contrary, it is called anti-Stokes Line (Jones et al., 2019). When photons collide with molecules, the energy between them does not change after the collision, but the direction is changed, which is called Rayleigh scattering (Bumbrah and Sharma, 2016). Normally, RS has a strong fluorescence background that could disturb the original spectrum, which leads to compromised quality of bacterial identification, although it could be removed by techniques like polynomial baseline fitting (Wei et al., 2015). As for the application, RS produces a series of spectral signal lines when measuring a particular sample, in which Raman shift is the frequency difference between the Raman scattered light and the aforementioned Rayleigh scattered light (Cialla-May et al., 2019). Some specific molecules in biological samples will have characteristic peaks, and the concentration or amount of a certain molecule in the sample will affect the intensity of the molecule (Figure 1).

## Bacterial Identification

Raman spectral features are generated by molecular vibrations in the sample, which makes RS a convenient tool for characterizing biological systems (Ashton et al., 2011). Due to its low-cost, label-free, and nondestructive features, RS has been widely investigated in terms of its potential applications in clinical studies. In addition, the sample preparation steps are simple, and the spectroscopic procedures can be completed within seconds, which makes it a promising method for detecting bacterial infection (Boardman et al., 2016). Through searching keywords RS and bacteria identification on the biomedical literature database, PubMed^1^, it has been observed that there is a continuously growing number of RS-assisted bacterial detection studies. However, there is still a hug gap between basic research and practical application, which prevents RS from becoming the routine laboratory technique. For example, Raman effect is very weak, which leads to long measurement times; moreover, sample fluorescence introduces noisy signals into the spectrum, which makes the downstream analysis rather difficult (Wei et al., 2015). In addition, intense laser radiation can cause sample heating, leading to sample destruction and disrupted Raman spectrum. Thus, biological samples should be investigated via low-energy near-infrared wavelength for excitation, e.g., 785 or 830 nm, or in water solutions (Eberhardt et al., 2015).

During bacterial sample analysis, RS provides information on both chemical compositions and biomolecular structures, such as DNA, RNA, proteins, lipids, and carbohydrates, which is often referred as whole-organism fingerprint (Ashton et al., 2011). For example, Raman signal for C–H stretching vibration is at approximately 2,930 cm^–1^ and C–H deformation vibration at approximately 1,440 cm^–1^, while the main signal for proteins is the amide I vibration at 1,665 cm^–1^ (Lorenz et al., 2017). In addition, RS has also been applied in single bacterial analysis and live bacterial studies, which could not only minimize the bacterial metabolic variability at the different phases but also facilitate the understanding of cellular dynamics (Strola et al., 2014; Smith et al., 2016). In terms of the differentiation of Gram-positive bacteria from Gram-negative bacteria, it was showed that some peaks at 540 and 1,380 cm^–1^ had significant differences for Gram-positive bacteria when compared with the Gram-negative bacteria, which was mainly attributed to the glycosidic bonds in *N*-acetyl glucosamine and *N*-acetyl muramic acid of peptidoglycan (de Siqueira E Oliveira et al., 2020).

So far, studies performing RS on clinical bacterial pathogens require culture in agar plates because of the low concentration of bacteria in clinical samples (Rebrošová et al., 2017; de Siqueira E Oliveira et al., 2020). Although culture-based RS could provide sufficient biomass during testing, hence higher signal–noise ratio, it is rather time consuming. There are also some attempts of RS applications on tissues in terms of infectious disease diagnosis *in situ*. Kloß et al. (2014) used RS and chemo-metrical evaluation to study the ascitic fluid directly for pathogen identification, which showed that 97.7% of the spectra from Gram-positive bacteria were correctly assigned on the genus level and 83.6% on the species level. In another study, Maquelin et al. (2003) used Raman spectra for rapidly identification of bacterial and fungal pathogens recovered from 115 blood cultures after 6- to 8-h culture in an automated blood culture system, according to which 109 samples contained bacteria while 6 contained yeasts (92.2% identification accuracy). Thus, RS possesses the potential in the identification of bacterial infections directly for clinical samples.

In some situations, clinical samples only contain trace amounts of bacterial cells. In order to improve the weak Raman signals in clinical samples such as blood and urine when bacterial amount is rather low, surface-enhanced Raman spectroscopy (SERS) can be applied, which could facilitate the development of culture-free identification of bacterial pathogens. In specificity, SERS is an enhanced RS through sample molecules interacting with surface plasmons of nanoscale structured metal surfaces, which often uses spherical nanoparticles made of silver or gold with diameters ranging from 20 to 100 nm (Krafft and Popp, 2014). For example, Tien et al. (2018) investigated bacterial pathogens in 108 urine samples sourced from of urinary tract infection patients; according to the study, 93 samples were detected with single bacterial species via SERS, while 97 samples were confirmed pathogen positive through medium culture, which makes the detection 95.87% accurate. Currently, although SERS is a highly promising analytical technique, it has not been used as a routine diagnostic method in the clinical laboratory yet, and there are many problems preventing its real-world application. One of the major limitations is the fabrication of suitable substrates with unique features in SERS-related detections, although tremendous effort has been invested into this area (Ouyang et al., 2017). Thus, developing new cost-effective and reproducible substrates for SERS would also greatly increase its sensitivity and accuracy, hence wider applications of the technique. Many studies have reported a variety of preparation procedures of nanoparticles for SERS (Solís et al., 2017; Demirtaş et al., 2020). However, this topic is rather large and is not a focus of the current review. For details, please refer to the recent review by Lane et al. (2015).

**FIGURE 2:**
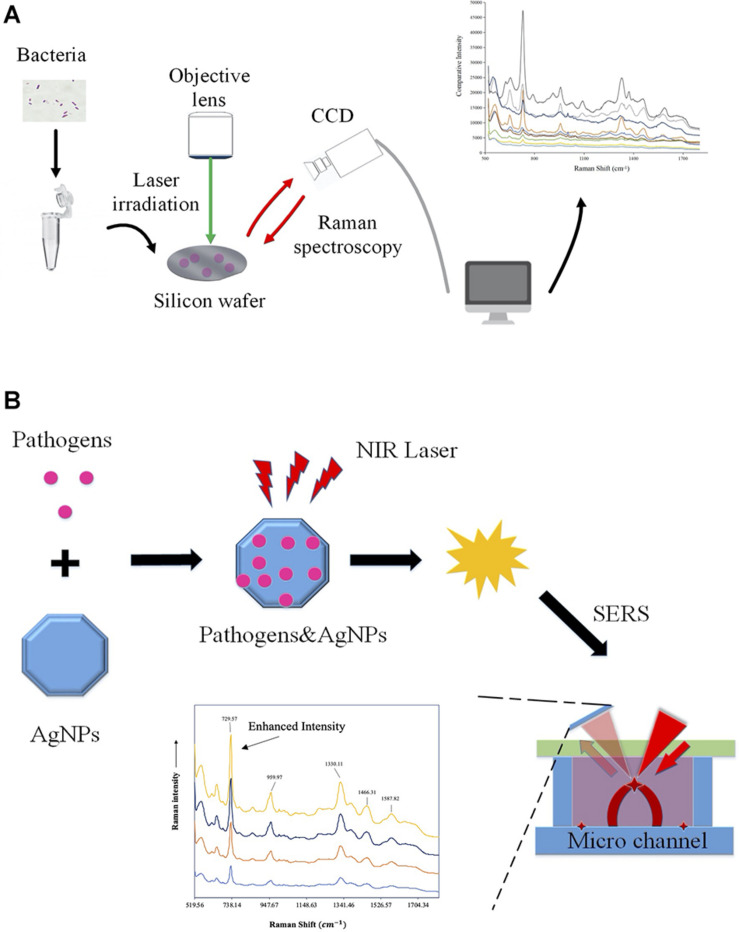
Schematic illustration of the workflow of Raman spectroscopy and surface-enhanced Raman spectroscopy (SERS). **(A)** Workflow of Raman spectroscopy obtaining information on molecular structure via molecular vibrations and rotations for bacterial classification and antibiotic resistance profiling. **(B)** Procedures of the surface-enhanced Raman spectroscopy. Samples were measured while being adsorbed on the surface of colloidal metal nanoparticles such as silver (AgNPs), gold, or copper in order to improve signal intensity.

Except for bacterial infections, RS has also been applied for the identification of other microbial species, which shows great promise for the accurate diagnosis of parasites and viruses (Chen et al., 2016; Yeh et al., 2020; Donald et al., 2021). In particular, since the global outbreak of coronavirus 2019 (COVID-19), a variety of studies focus on the rapid detection of severe acute respiratory syndrome coronavirus 2 (SARS*-*CoV*-*2) via RS. For example, Carlomagno et al. (2021) reported a Raman-based method for saliva analysis, which is able to differentiate healthy individuals from infected patients with accuracy, precision, sensitivity, and specificity of more than 95%. In addition, Yin et al. (2021) analyzed 177 serum samples (63 confirmed COVID-19 patients, 59 suspected cases, and 55 healthy individuals) via RS, together with 20 independent individuals for external validation. According to the study, accuracy between the COVID-19 and the healthy controls is 0.90, which also indicated that RS held the promise of being a safe and efficient technique for COVID-19 screening (Yin et al., 2021). For a schematic illustration of the workflow of Raman spectroscopy and surface-enhanced Raman spectroscopy (SERS), please refer to Figure 2.

## Antibiotic Resistance Profiling

As for AST, it is an essential procedure in the clinical diagnosis of serious bacterial infection, while accurate and effective diagnosis of bacterial antibiotic resistance is a key for the treatment of bacterial infections (Wang et al., 2018). Although the typical procedure normally takes 3–4 days or even longer for fastidious bacteria on average to obtain the final AST results (Han et al., 2020), with MALDI-TOF MS-based approaches, i.e., for positive blood culture bottles, a result can be obtained after <24 h, in some cases also the same day (Verroken et al., 2014). Due to its simple operations, RS, especially SERS, has been used for testing antibiotic resistance phenotypes in many bacterial species, such as *E. coli* (Chang et al., 2019), *S. aureus* (Uysal Ciloglu et al., 2020), and *Pseudomonas aeruginosa* (Li et al., 2019). A variety of signatures have been observed in terms of bacterial antibiotic resistance and susceptibility, which could be used for rapidly identifying resistance to sublethal concentrations of antibiotics (Galvan and Yu, 2018; Han et al., 2020). In addition, a single study also reported that a portable Raman spectrometer with paper-based SERS could be used for screening tetracycline residues in milk with peak intensity ratios at 455 cm^–1^/1,280 cm^–1^ and 874 cm^–1^/1,397 cm^–1^. Thus, RS could function as a potential tool for on-site monitoring of antibiotics (Marques et al., 2019). However, despite that SERS was investigated to detect antibiotic-resistant phenotypes in some studies, current datasets are small, limited, and often involving environmental settings. In addition, the ability of RS to detect resistance phenotypes is something different from antibiotic resistance testing, which does not rely on the presence of resistance markers but on the determination of minimum inhibitory concentration (Galvan and Yu, 2018). Thus, the generalization of these Raman signatures, biomarkers, or metabolites in predicting antibiotic resistance profiles should be further examined before applied in clinical settings.

## Computational Analysis of Raman Spectra

Due to the complexity of Raman spectra, statistics and machine learning algorithms, rather than traditional linear analysis, are normally involved in data processing procedures. So far, many machine learning methods have been introduced into Raman spectra analysis, such as artificial neural network, deep learning, and Monte Carlo estimation (Lu et al., 2012; Moawad et al., 2019; Lussier et al., 2020; Uysal Ciloglu et al., 2020). In the rapid characterization of *Staphylococcus*, Rebrošová et al. (2017) compared three machine learning methods, namely, linear discriminant analysis, one nearest neighbor, and support vector machine (SVM), all of which showed efficient identification of staphylococci using RS with high accuracy. Although machine learning often gives promising results during the analysis of Raman spectra, there are some particular pitfalls that should be avoided. The Raman spectra dataset should be large enough for the training and validating steps in order to make sure that the learning process is sufficient. In addition, collection of Raman spectral data is more important than models and algorithms themselves since over- or underrepresented data will lead to biased predictions. Moreover, what machine learning algorithms to choose and how the mode parameters are determined are also crucial for Raman spectral analysis.

## Raman Spectral Database of Bacterial Pathogens

A precondition for using machine learning to analyze Raman spectral data is a database with validated reference spectra of bacterial species and phenotypes (Moawad et al., 2019). It is convenient to measure single bacterial spectra from cultures in the lab, which is normally crucial to build a preliminary Raman spectral database. However, in order for the database to be functional in real-life environment, a database of Raman spectra from environmental or patient samples is required (Pahlow et al., 2015). Raman spectral databases have been constructed in a variety of fields, such as minerals, organics, inorganics, essential oils, pigments, and carbohydrates, which greatly facilitates the detection of these materials and further increases the applications of RS in corresponding fields (Strola et al., 2014; Kumar et al., 2015; El Mendili et al., 2019). Thus, a standard database of Raman spectra for bacterial pathogens would be very convenient and highly demanded in species identification and antibiotic resistance profiling.

Lorenz et al. (2017) emphasized the importance of Raman microscopic databases in the identification of leading pathogens in environmental and patient samples. Strola et al. (2014) constructed a reference database including a total of 1,200 spectra over seven bacterial species, based on which the success rate of bacterial species identification approaches 87% via SVM classification. Kloß et al. (2014) built up a Raman database containing 10,000 single-cell spectra for 34 bacterial strains belonging to 13 different species in ascitic fluids. In addition, Novelli-Rousseau et al. (2018) tried to distinguish antibiotic-resistant and antibiotic-susceptible *E. coli* based on a database with 3,668 Raman spectra. Some other studies also implement several small bacterial Raman databases that greatly promote the application of RS in bacterial analysis (Muhtar et al., 2016).

Unfortunately, at current stage, there is very little effort dedicating to the integration of small database into a large and standard Raman spectral database in bacterial field that may be widely used in different microbiological and clinical labs. A particular reason for such a deficiency is that Raman spectra from different studies are tailor-made and group specific, which greatly hinders data standardization (Lorenz et al., 2017). In order to facilitate the standardization of RS data, metadata annotation with minimal sample preparation and acquisition of Raman spectra is indispensable, which could not only alleviate technical noisy signals but also improve reproducibility in RS experiments (García-Timermans et al., 2018). Furthermore, sample preparation recommendations and data-processing guidelines should be introduced for future work, which shall greatly promote the application of RS and translate it into a routine diagnostic method in clinical laboratory.

## Summary

Raman spectroscopy can provide bacterial phenotypic information in details and vast amounts. Although numerous studies focus on rapid identification of bacterial species and antibiotic resistance profiles by RS, the real situation is that the technique has not been fully explored in clinical settings yet. Currently, most Raman spectra of bacterial pathogens are based on pure bacterial isolates, which heavily relies on medium culture, while Raman spectra from actual clinical samples are still rare. Recently, with the development of nanoparticles and nanostructured surfaces, SERS greatly improved the signal intensity of Raman spectra, which greatly contributes to a better differentiation of bacterial infections. In addition, Raman spectra consist of the spectra of a large set of complex chemical mixtures, requiring machine learning methods for data processing, such as artificial intelligence and deep learning, rather than classical linear methods. However, problems encountered during machine learning-assisted analysis involve overfitting or underfitting of the models due to the large dimension and small sample size problem of Raman spectra, although there are different dimension reduction methods like principal component analysis in use to address the issue. In addition, standard database of RS for bacterial pathogens is also a guarantee of the accurate and timely laboratory diagnosis when recruiting machine learning methods. In sum, the techniques of rapid and reliable automatic measurement of the Raman spectra of clinical samples from the real word are eagerly and urgently needed for the applicability of bacterial typing and antibiotic resistance profiling in clinical settings, which shall be achieved in foreseeable future with the fast development of novel Raman spectroscopic techniques, nanostructural materials, computational methods, and standardized databases.

## Author Contributions

LW conceptualized the manuscript. LW, XZ, and BG contributed to project administration. LW, WL, J-WT, Q-HL, BG, and XZ wrote the original manuscript. LW, WL, P-BW, and XZ provided platform, resources, and student supervision. J-JW, J-WT, M-MW, and Y-CP revised the manuscript. All authors read and approved the final manuscript.

## Conflict of Interest

The authors declare that the research was conducted in the absence of any commercial or financial relationships that could be construed as a potential conflict of interest.

## Footnotes

1 https://pubmed.ncbi.nlm.nih.gov/
